# Sleep in Chronic Obstructive Pulmonary Disease: Evidence Gaps and Challenges

**DOI:** 10.1155/2016/7947198

**Published:** 2016-06-08

**Authors:** Rachel Jen, Yanru Li, Robert L. Owens, Atul Malhotra

**Affiliations:** ^1^Clinical Investigator Program, Department of Medicine, University of British Columbia, Vancouver, BC, Canada V5Z 1M9; ^2^Division of Pulmonary, Critical Care and Sleep Medicine, University of California, San Diego, La Jolla, CA 92037, USA; ^3^Department of Otorhinolaryngology-Head and Neck Surgery, Sleep Medicine Center, Beijing Tongren Hospital, Capital Medical University, Beijing 100730, China

## Abstract

Chronic obstructive pulmonary disease (COPD) prevalence is rising to epidemic proportions due to historical smoking trends, the aging of the population, and air pollution. Although blaming the victims has been common in COPD, the majority of COPD worldwide is now thought to be nonsmoking related, that is, caused by air pollution and cookstove exposure. It is increasingly appreciated that subjective and objective sleep disturbances are common in COPD, although strong epidemiological data are lacking. People with obstructive sleep apnea (OSA) plus COPD (the so-called overlap syndrome) have a high risk of cardiovascular death, although again mechanisms are unknown and untested. This review aims to draw attention to the problem of sleep in COPD, to encourage clinicians to ask their patients about symptoms, and to stimulate further research in this area given the large burden of the disease.

## 1. Background

Recent data suggest that roughly 10% of the population over the age of 40 years has clinically important chronic pulmonary obstructive pulmonary disease (COPD), with the majority of patients remaining undiagnosed and untreated [1]. Sleep is a period of vulnerability for people with COPD for a number of reasons. First, the onset of sleep represents loss of the so-called “wakefulness drive to breathe” such that COPD patients can experience deterioration in gas exchange. Hypercapnic chemosensitivity is also reduced such that, for a given change in CO_2_, the increase in ventilation is minimal in people with COPD during sleep [2]. Patients with COPD can experience profound desaturation instances during rapid eye movement (REM) sleep in part related to atonia in the skeletal muscles including the accessory muscles of respiration. Second, cough is typically suppressed during sleep such that people with COPD can develop mucus plugging and hypersecretion, affecting nocturnal gas exchange. Lack of cough overnight often leads to productive cough in the morning, which can be disabling. Third, obstructive sleep apnea (OSA) as discussed below is a common disorder which can occur in COPD patients: the concurrence of these two conditions is known as overlap syndrome which is associated with a poor prognosis [3]. Thus, a strong physiological basis exists for why COPD patients have poor sleep quality.

## 2. Sleep Complaints

Research indicates that more than 60% of patients with COPD experience sleep symptoms and/or bothersome dyspnea/cough at night, although these complaints are often underreported by patients and are not part of routine clinical management [4, 5]. The nature of these complaints is quite variable and can be nonspecific but includes symptoms like insomnia, nonrestorative sleep, daytime fatigue, and nocturnal cough. In addition, other sleep disorders are common in COPD patients. For example, one-third of COPD patients are estimated to have restless leg syndrome, which can further negatively affect sleep quality [6]. Although patients with mild COPD have relatively preserved sleep quality, severe disease is associated with objectively measured worse sleep quality, including decreased total sleep time, decreased sleep efficiency, and increased sleep fragmentation. Poor sleep quality in COPD predicts subjective health-related quality of life (HRQoL) [4], and poor sleep has been associated with adverse outcomes including exacerbation and hospitalization [7, 8]. Therefore, nocturnal and sleep symptoms should be part of the routine clinical evaluation for COPD patients and this often forgotten aspect of COPD care warrants further clinical investigation. Medications such as sedative hypnotics are often used to treat sleep-related symptoms but these medications could at least in theory reduce respiratory drive and worsen hypoxemia; nonsedative hypnotics such as ramelteon (a melatonin receptor agonist) might be a safer alternative [9]. Some recent data have shown that pulmonary rehabilitation can lead to improved sleep quality although it remains unclear which component of rehabilitation is leading to the observed improvement [10]. In theory, improved overall wellbeing with rehabilitation including exercise performance and motivation to undertake daytime activities may play a role in improvements in sleep quality.

## 3. COPD and Sleep Apnea: The Overlap Syndrome

There is controversy regarding whether sleep apnea prevalence is higher in COPD as compared to matched individuals without COPD. The data suggest highly variable prevalence figures depending on the severity of COPD, the diagnostic techniques employed, and the associated risk factors in the cohort being studied. For example, obesity is a major risk factor for sleep apnea, but prevalence of obesity is variable in COPD depending on COPD severity, particularly since some emphysema patients experience cachexia related to underlying illness. Similarly, systemic glucocorticoids use can promote weight gain and thus increase OSA risk, but their use may improve dynamic hyperinflation and nocturnal oxygenation in these patients. End-expiratory lung volume may be an important factor since increased lung volume has been associated with upper airway stability [11], but, in theory, loss of lung elastic recoil in emphysema may diminish the caudal traction forces important in promoting upper airway patency. In the aggregate, the data suggest no major increase in risk of OSA in people with mild-to-moderate COPD but a potentially higher risk of OSA in severe COPD. Indeed, a study by Soler et al. reported 66% prevalence of OSA in moderate-to-severe COPD patients, a finding which is quite striking when one considers the large worldwide burden of COPD [12]. However, the association of the sleep apnea and COPD disease severity is not clearly defined at this point. A recent paper from the COPDGene study showed high prevalence of OSA in the severe COPD group but the AHI was inversely correlated with degree of emphysema and hyperinflation of the CT scans in the patients with overlap syndrome [13]. Another study by Biselli el al. confirmed this finding by demonstrating that hyperinflation of COPD is associated with more stable upper airway, which is measured by critical closing pressure (Pcrit) during sleep [11]. More research is definitely needed to stratify the risk of OSA in COPD patients. Furthermore, untreated OSA in overlap syndrome is associated with increased mortality and risk of COPD exacerbation [3], leading to worse prognosis [14]. On the other hand, the potential reversibility of cardiovascular risks with CPAP treatment in overlap syndrome supports a more aggressive approach to identify OSA in COPD.

## 4. Diagnosis of Breathing Disorders during Sleep in COPD

Nocturnal hypoxemia is a common sleep abnormality in COPD patients, and it is estimated to affect 27–70% of COPD patients with awake resting oxygen saturation of 90–95% even without any upper airway obstruction [15, 16]. Nocturnal hypoxemia can be due to sleep apnea (obstructive, central, or mixed), hypoventilation due to underlying COPD, or combination of all of them. Nocturnal hypoxemia has been associated with increased mortality and exacerbation in COPD but optimal management of nocturnal desaturation remains uncertain pending the results of large ongoing multicenter trials (e.g., NCT01044628 [17] and NCT00692198 [18]). Therefore, it is important to evaluate nocturnal oxygen saturation in COPD patients at risk (with saturation less than 95% during wakefulness) for prognostication. Nocturnal hypoxemia can be evaluated by polysomnography or portable sleep testing with various sensors. Nocturnal oximetry is one of the simplest portable sleep testing methods with two channels (heart rate and oximetry), but nocturnal oximetry alone has limited utility as a diagnostic tool for OSA in COPD patients because it cannot effectively differentiate nocturnal hypoxemia from COPD or OSA, to which different treatment approaches apply. Thus, polysomnography with transcutaneous CO_2_ monitoring is the current best tool to evaluate sleep disordered breathing for COPD patients. The role of unattended portable sleep study in this population is unclear and more research is needed. As untreated overlap syndrome patients carry a poor prognosis and CPAP may eliminate the additional mortality risk from untreated OSA, clinicians should have a high index of suspicion for the following patients: (1) patients with sleep complaints or symptoms suggestive of OSA; (2) patients with mild COPD and evidence of pulmonary hypertension or daytime hypercapnia; (3) COPD patients with nocturnal oxygen desaturation who developed morning headaches when treated with nocturnal supplemental oxygen.

Of note, the conventional metric of sleep apnea severity, the apnea hypopnea index (AHI), may be more difficult to interpret in the setting of chronic lung disease and hypoxemia. For instance, a patient who experiences a prolonged period of hypoventilation would typically be counted as having single hypopnea without transcutaneous CO_2_ monitoring, despite the likelihood that this event has the potential for deleterious consequences. In addition, supplemental oxygen during diagnostic PSG can clearly reduce hypoxemia but may also reduce the diagnostic sensitivity of the pulse oximeter given the shape of the oxyhemoglobin saturation curve. Nasal pressure transducer signal is often degraded when supplemental oxygen is administered due to the local flow effects on the pressure transducer. Also, the concurrent use of nasal cannula for supplemental oxygen, nasal pressure transducer, and oronasal thermal sensor often causes discomfort for patients during PSG given the limited space inside the nostrils. Lastly, transcutaneous CO_2_ is an important surrogate measure for arterial carbon dioxide pressure in COPD patients to evaluate sleep-related hypoventilation. However, transcutaneous CO_2_ cannot provide breath-by-breath CO_2_ changes and may be misleading at times. Due to these technical difficulties, differentiation between REM-related hypopnea and hypoventilation, which may have different treatment indications, is often challenging in COPD patients. Thus, at present, traditional sleep apnea diagnostic criteria are valuable for the OSA diagnosis, but further research in developing OSA indices is encouraged to properly define disease burden and consequences.

## 5. Mechanism of Increased Cardiovascular Mortality of Overlap Syndrome

A study recently reported that the mass of the right ventricle is increased in overlap syndrome patients compared to matched individuals with COPD alone [19]. This load on the right ventricle presumably reflects hypoxic pulmonary vasoconstriction coupled with dilation and destruction of the lung parenchyma in emphysema. The clinical significance of the right ventricular abnormalities is unclear but may provide insight into why overlap syndrome patients are at high risk of cardiovascular events. In addition, using novel cardiac MRI methods, the degree of myocardial fibrosis in people with COPD was quantified and an increase in the degree of myocardial fibrosis was found to be associated with degree of hypoxemia, a finding which may explain in part the impairment in exercise performance in these individuals [20]. These pilot studies [19, 20] provide some insights into the potential mechanism but should not be regarded as definitive: further work regarding cardiovascular function in COPD is clearly needed.

## 6. Treatment: Continuous Positive Airway Pressure (CPAP) versus Noninvasive Ventilation (NIV)

**Figure 1:**
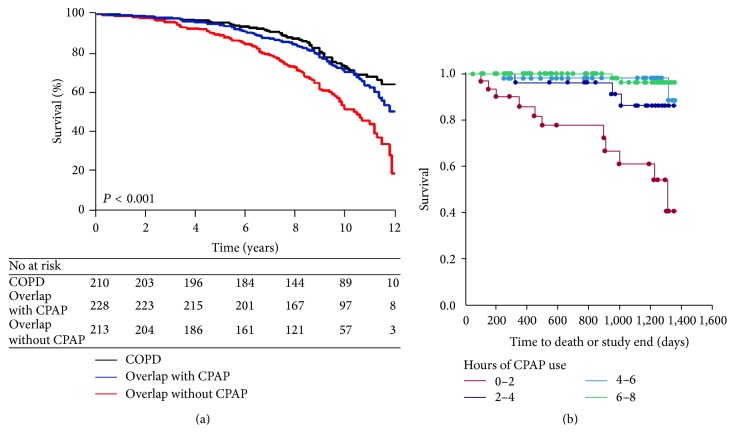
(a) Kaplan-Meier survival curves for survival among COPD patients, overlap syndrome patients without CPAP treatment, and overlap syndrome patients on CPAP treatment. This study included only severe OSA patients (AHI > 30), and CPAP was not randomly assigned (from [3], permission granted); (b) Kaplan-Meier survival curves for survival among patients with COPD and OSA overlap syndrome, stratified by CPAP use per night (from [22], permission granted).

Two large observational cohorts have demonstrated significant mortality benefit with CPAP (continuous positive airway pressure) treatment in overlap syndrome patients compared to medical therapy (see Figure 1(a)) [3, 21]. Moreover, a study has shown a dose related improvement in outcome as a function of CPAP use, such that each extra hour of CPAP use was associated with improvements in survival (see Figure 1(b)) [22]. Adherence to CPAP may be a marker of a good prognosis (“healthy user effect”) emphasizing the need for randomized trials in this area. The use of oxygen is recommended for COPD patients with daytime hypoxemia but benefits of long-term oxygen therapy for nocturnal hypoxemia have been lacking [23]. However, the optimal treatment of overlap syndrome patients remains unclear but may well require a combination of supplemental oxygen and positive airway pressure therapy. The use of noninvasive ventilation (NIV) in acute exacerbation of COPD has been well established, but the long-term nocturnal use in stable COPD has been more controversial. A number of studies have been published in this area with many showing no major benefit with bilevel positive airway pressure (BPAP).

One recent study by Köhnlein et al. [24] has received considerable attention. The authors in Germany and Austria randomized hypercapnic COPD patients to receive BPAP or usual care to assess the impact on mortality. The authors did observe an improvement in mortality using BPAP, associated with improvement in hypercapnia; however, the generalizability of this study has been questioned. For example, very few hospitalization cases were observed in the Köhnlein study despite a large number of deaths suggesting practice deviation from North America where many severe COPD patients undergo recurrent hospitalization before succumbing. High-intensity NIV has also received attention. Windisch et al. [25] have generated an important body of literature showing that large inspiratory pressures (up to 30 cm H_2_O) can be used safely in COPD and may lead to improvements in respiratory function and various outcome measures. The viability of this approach is being tested by other groups currently.

**Figure 2:**
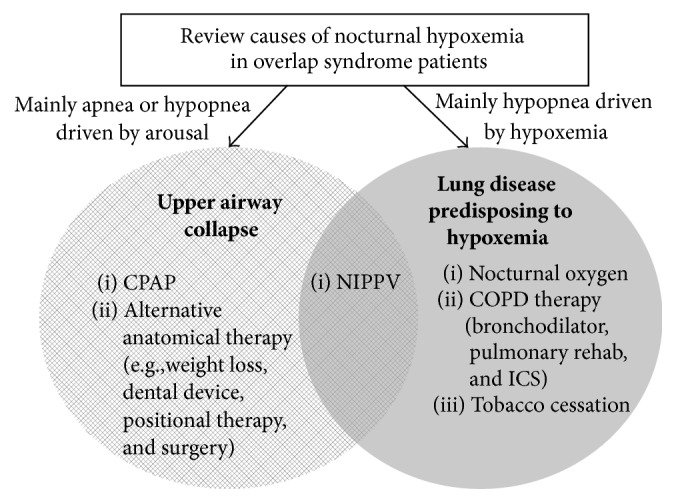
Treatment options for patients with COPD and OSA overlap syndrome. CPAP: continuous positive airway pressure; NIPPV: nasal intermittent positive pressure ventilation; ICS: inhaled corticosteroids.

Despite the encouraging results, the optimal treatment of patients with overlap syndrome remains undefined. One often overlooked therapy for overlap syndrome is treatment for COPD including bronchodilator and steroid therapy, showing improved nocturnal hypoxemia in overlap syndrome patients. In theory, treatments targeting hypoxemia and/or hypercapnia can be used separately or in combination to achieve the optimal goal: normal gas exchange in COPD patients (see Figure 2) [26]. Further studies are clearly needed.

## 7. Summary

In summary, further efforts regarding issues with sleep in COPD are urgently needed. Clinicians should be aware of the high prevalence of sleep abnormalities in COPD, many of which can change management if addressed appropriately. A careful history can be helpful in defining the nature of the abnormality although objective testing is frequently required to draw conclusions. However, many unresolved questions from a mechanistic as well as clinical standpoint remain, emphasizing the need for further study. To date, many OSA studies have excluded COPD patients and many COPD studies have either ignored or excluded OSA. Thus, we all have work to do.
